# Predictors of Neonatal Intensive Care Unit Admission and Adverse Outcomes Related to Gestational Diabetes

**DOI:** 10.7759/cureus.38579

**Published:** 2023-05-05

**Authors:** Abdullah M Al-shahrani

**Affiliations:** 1 Family Medicine, College of Medicine, University of Bisha, Bisha, SAU

**Keywords:** adverse outcomes, neonate, admission, predictors, gestational diabetes

## Abstract

Background: Gestational diabetes mellitus (GDM) is a type of diabetes that manifests itself in pregnant women. It poses a significant risk to the mother’s health as well as the health of the infant, including more babies being brought to the neonatal intensive care unit (NICU). It puts both the mother's and the child's health at serious risk, increasing the likelihood that newborns may need to be treated in a neonatal critical care unit. This study aimed to determine the factors that predict GDM-related NICU admission and other adverse newborn outcomes.

Methods: The study was a cross-sectional analysis of 175 pregnant women who presented with gestational diabetes at the Maternity and Children’s Hospital in Bisha (MCH-Bisha), Saudi Arabia, between January 1 and December 31, 2022. A logistic regression model was used to analyze the data to predict adverse outcomes for newborns and NICU admissions and identify associations between maternal variables and outcomes.

Results: Maternal characteristics highly associated with adverse neonatal outcomes included advanced maternal age (greater than 30 years), a family history of DM, and a history of greater than or equal to four previous pregnancies. Logistic regression models revealed that newborns delivered to mothers older than 30 years were 7.17 times more likely to be admitted to the NICU than newborns born to mothers younger than 30 years. Saudi nationality, urban residence, and cesarean section delivery factors account for nearly all adverse neonatal outcomes (91%, 75%, and 91%, respectively). Newborns delivered by cesarean section were 3.38 times more likely to be admitted to NICU, and the association was significant.

Conclusions: Maternal age greater than 30 years and a history of more than or equal to four pregnancies were the strongest indicators of infant adverse outcomes and NICU admittance among women with gestational diabetes. These findings highlight the need for approaches to GDM management that are efficient, thorough, and multidisciplinary.

## Introduction

Gestational diabetes mellitus (GDM) is a form of diabetes that develops in pregnant women. It poses a significant risk to the mother’s health as well as the health of the infant. Insulin is a protein hormone that is too large to pass through the placenta from the mother to the fetus. Therefore, insulin produced by the mother's pancreas cannot directly affect the fetus in cases of GDM [1]. The chance of GDM is raised by a number of variables, including but not limited to a family history of diabetes, and an old mother according to age [2].

GDM had a 14.0% aggregated worldwide standardized prevalence. The Middle East and North Africa (MENA) had the greatest average prevalence of 27.6%. Women in the (MENA) area are at risk for GDM because of things like obesity and excessive parity. The Gulf Cooperation Council (GCC) nations had the greatest prevalence (14.1%). Saudi Arabia had the greatest GDM nationwide (21.6%) [3,4].

Admitting an infant to the NICU is expensive and has more negative consequences for the baby, his family, and the community at large. So, preventing such events is important from the health system's and families' perspective regarding cost, risks, and stressful moments. [5]. In term neonates born to women with GDM, the risk for NICU admission revealed that these infants had lower birth weights, increased fetal growth restriction (FGR) risk, decreased Apgar ratings at 5 min, and increased respiratory distress syndrome (RDS) incidence [6]. Studies have linked GDM to an increase in NICU admission rates as well as negative consequences on both mothers and newborns [7-9].

In a population with a high prevalence of type 2 DM, a two-year study in New Zealand revealed an increase in NICU admissions related to neonatal morbidity, which was prevalent in neonates born to mothers with type 2 DM and GDM [10]. Studies from Saudi Arabia and Iraq have shown that older mothers are more likely to have babies admitted to NICU due to GDM than younger mothers. This is likely due to an increased risk of developing GDM as well as other medical complications associated with older age [11,12].

GDM mothers were considerably more likely to experience RDS, hypoglycemia, macrosomia, NICU admission, hyperbilirubinemia, birth trauma, and premature birth than non-GDM mothers [13]. GDM risk factors were identified in women from a low-to-middle socioeconomic population who were older, had a higher body mass index (BMI), and had pre-diabetes [14].

Gestational diabetes increases the likelihood that a neonate will be admitted to the NICU, have a premature birth, be induced into labor, or be delivered by a method other than spontaneous vaginal delivery (SVD) [15]. Babies born from mothers with GDM may experience adverse outcomes such as macrosomia (large birth weight), hypoglycemia (low blood sugar), RDS, jaundice, and birth trauma such as shoulder dystocia or clavicular fracture from difficult delivery [16,17].

Around the globe, gestational diabetes is a common pregnancy challenge. It is linked to a higher chance of adverse outcomes for the mother and the child. Healthcare professionals can better handle and treat pregnant women with gestational diabetes by identifying indicators of these negative consequences, possibly lowering the risk of problems. The admission of a child to the NICU is a big deal for the family and can be difficult financially and emotionally. Studying predictors of adverse outcomes related to gestational diabetes can provide evidence for health decision-makers aimed at reducing the incidence and impact of this condition. By identifying modifiable risk factors associated with adverse outcomes, interventions can be developed to improve maternal and neonatal health outcomes. This study aimed to determine what variables contributed to GDM-related NICU admission and other adverse newborn outcomes.

## Materials and methods

Study design setting

This cross-sectional study was done on women with gestational diabetes who presented to the Maternity and Children’s Hospital, Bisha (MCH-Bisha) in Southern Saudi Arabia. The time period covered by the study was from January 1, 2022, to December 31, 2022, from recruitment to completion. The Institutional Review Board of the University of Bisha provided ethical clearance (UBCOM/H-06-BH-087, 06/26). The relevant institutions were informed, and their permission was obtained accordingly.

Study population

Women with diabetes were recruited from the 3550 healthy deliveries at the hospital during the study period. One hundred seventy-five women who were diagnosed with gestational diabetes and matched the inclusion criteria were recruited from the hospital records database.

Prenatal care was initiated in primary care settings, with screening for gestational diabetes beginning earlier in pregnancy for patients at high risk. Routine screening is performed on all pregnant women between 24 and 28 weeks of pregnancy. A glucose challenge test (GCT) is the initial stage in the screening procedure, which includes ingesting a glucose solution and then having blood collected an hour later to measure blood sugar levels. A diagnostic oral glucose tolerance test (OGTT) is conducted if the result is abnormal, with a blood sugar level of 140 mg/dL or greater.

According to the Ministry of Health's standards, which were adopted from the World Health Organization (WHO) guidelines, all patients with abnormal results were sent to the Maternity and Children's Hospital for further investigation and confirmation using the OGTT [18]. The diagnosis of GDM is based on meeting one or more of the following criteria: fasting plasma glucose levels ranging from 5.1 to 6.9 mmol/L (equivalent to 92-125 mg/dL); 1-hour post-75-g oral glucose dose showing glucose levels of 10 mmol/L (equivalent to 180 mg/dL); 2-hour post-75-g oral glucose dose showing glucose levels of 8.5-11.0 mmol/L (equivalent to 153-199 mg/dL).

After being given the initial diagnosis of gestational diabetes by primary health care (PHC) physicians, they began antenatal care sessions with a consultant-led, interdisciplinary team. All hospital deliveries were overseen by a pediatrician, who performed an instant assessment of the baby and made decisions about the next steps in the infant’s care based on the results of that assessment. Women whose primary care providers recommended delivering their infants at the MCH hospital and were identified with GDM were eligible to participate in this research. Exclusion criteria included having more than one pregnancy, lacking or insufficient medical documentation, having a history of diabetes (type 1, type 2, or prior GDM), not receiving prenatal care in the hospital, experiencing complications, and having incomplete records.

As part of managing gestational diabetes, glucose levels were monitored for every woman. Women's blood sugar levels were monitored throughout pregnancy, and their insulin dosage was adjusted as necessary. Women who cannot control their blood sugar levels through diet and exercise are administered insulin therapy. Close monitoring and treatment were done to promote lung maturation through the use of prenatal corticosteroids, particularly in cases of premature birth or suspected cases. A cesarean section was considered if the fetal weight exceeded 4 kg or if there were repeated cesarean sections and other medical conditions that make a vaginal delivery risky.

Adverse neonatal outcomes definitions

Pediatric specialists attended the delivery and assessed the neonates immediately at birth. The final diagnosis is after doing all the necessary physical examinations, blood tests, and radiology investigations. Infants were considered to be in respiratory distress when they displayed symptoms like breathing difficulties, cyanosis, and a change in their respiration rate. Hypoglycemia occurs when a newborn’s plasma glucose level falls below 40 mg/dL within 24 hours of birth. It was decided that an infant weighing more than 4 kg was considered to be macrosomic. A low-birth-weight infant is one whose initial weight is less than 2 kg. A newborn with an abnormality of the heart or the major arteries is referred to as having congenital heart defects (CHD) [19-21].

Data collection and management

The sociodemographic and general features of the women (age, residency, nationality, family history, number of previous pregnancies, and mode of delivery) were the maternal factors used in the study from the hospital records database to align with the study objectives. Also, other information related to adverse neonatal outcomes and admission to the NICU was collected. An Excel master sheet was used to gather the information and then transferred to the STATA program for analysis. Beginning with a system data review, we compared it to records from the delivery area, newborn unit, and diabetes clinic registry. The data was then handled to ensure the accuracy of the documented variables and to look for any potential absent variables.

Data analysis

STATA/BE version 17.0 (College Station, TX: StataCorp LLC) was used for all statistical testing and analysis. The command summary was utilized for descriptive statistics, and the command tabulates with the chi-square test were utilized to identify significant associations. The univariate logistic regression model underwent additional analysis to determine whether it could predict adverse newborn outcomes and NICU admittance (odds ratio, p-value, and 95% CI). A p-value of 0.05 or less was regarded as statistically significant.

## Results

The study included 175 pregnant women with a diagnosis of gestational diabetes. Most of the women were over the age of 30 years (56%), Saudi individuals (92.6%), and living in urban areas (81.7%). About 6.3% of women had a history of diabetes in their families. Also, as shown in Table 1, a large percentage of these mothers (78.3%) have already given birth via cesarean section, and 25.1% have had four or more births in the past.

**Table 1:** General characteristics of the study sample (n=175).

Variables		n (%)
Age (in years)	<30	77 (44)
	≥30	98 (56)
Nationality	Saudi	162 (92.6)
	Non-Saudi	13 (7.4)
Residence	Urban	143 (81.7)
	Rural	32 (18.3)
Family history of DM	Yes	11 (6.3)
	No	164 (93.7)
Number of previous pregnancies	<4	131 (74.9)
	≥4	44 (25.1)
Mode of delivery	Vaginal	38 (21.7)
	Cesarean section	137 (78.3)

**Figure 1:**
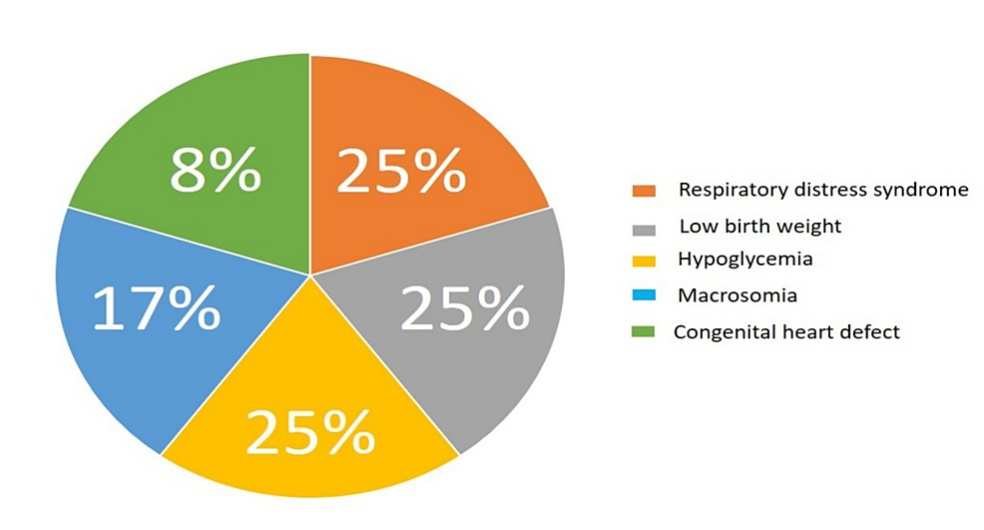
Adverse outcomes among infants delivered to women with GDM (n=12). GDM: gestational diabetes mellitus

Figure 1 and Table 2 illustrate the maternal characteristics associated with adverse infant outcomes. Twelve (7%) babies born to mothers with GDM had health problems like RDS (25%), low birth weight (25%), macrosomia (25%), hypoglycemia (17%), and heart defects (8%). All these babies with these complications were admitted to the NICU. They were discharged after various durations (from two weeks to three months) except for one neonatal death with multiple congenital anomalies (heart, neural tube, and limb abnormalities) diagnosed in late pregnancy due to delayed referral.

**Table 2:** Maternal characteristics and adverse neonatal outcomes among women with GDM (n=175). GDM: gestational diabetes mellitus

Variables		Adverse neonatal outcomes	Adverse neonatal outcomes	p-Value
		No (n=163)	Yes (n=12)	
Age (in years)	<30	76	1	0.01
	≥30	87	11	
Nationality	Saudi	151	11	0.91
	Non-Saudi	12	1	
Residence	Urban	134	9	0.53
	Rural	29	3	
Family history of DM	Yes	8	3	0.05
	No	155	9	
Number of previous pregnancies	<4	128	3	0.01
	≥4	35	9	
Mode of delivery	Vaginal	37	1	0.24
	Cesarean section	126	11	

Women over the age of 30 years (p=0.01), a family history of diabetes (p=0.05), and those with a history of more than or equivalent to four births in the past (p=0.01) were more likely to experience the adverse neonatal outcome. However, the study found no statistically significant link between being a Saudi individual, living in an urban area, and giving birth by cesarean section, despite these factors accounting for nearly all adverse outcome events (91%, 75%, and 91%, respectively).

The results of a logistic regression analysis that looked at each predictor and potential associations between GDM and newborn intensive care unit admission are presented in Table 3. If the mother was older than 30 years, there was a greater chance that the baby would be admitted to an intensive care unit (OR 7.17, p=0.001, 95% CI: 2.83-18.15). There were 1.09 times as many Saudi residents as non-Saudis admitted to the NICU (OR 1.09, p=0.89, 95% CI: 0.28-4.16). Births to mothers living in cities had 1.2% higher odds of being admitted to the NICU than those born to mothers living in rural regions (OR 1.2, p=0.69, 95% CI: 0.47-3.01). There was a highly statistically significant association between having more than four pregnancies and having a baby admitted to the NICU (OR 2.9, p=0.004, 95% CI: 1.39-6.16), and the odds ratio (OR) for developing DM among women with a history of DM in their family was 1.16 (p=0.83, 95% CI: 0.29-4.59) compared to women without such a history. There was a significantly increased risk of newborn intensive care unit admission for babies born via cesarean section (OR 3.38, p=0.03, 95% CI: 1.12-10.16).

**Table 3:** Logistic regression analysis for predictors and potential associations between GDM and newborn intensive care unit admission (n=175). GDM: gestational diabetes mellitus

Variables	Odds ratio	p-Value	95% confidence interval
Age (≥30 versus <30 years)	7.17	0.001	2.83-18.15
Nationality (Saudi versus non-Saudi)	1.09	0.89	0.28-4.16
Residence (urban versus rural)	1.2	0.69	0.47-3.01
Family history of DM (yes versus no)	1.16	0.83	0.29-4.59
Number of previous pregnancies (≥4 versus <4)	2.9	0.004	1.39-6.16
Mod of delivery (cesarean section versus vaginal)	3.38	0.03	1.12-10.16

Table 4 displays the outcomes of a logistic regression analysis that examined potential predictors and associations between gestational diabetes and adverse infant outcomes. Newborns of women aged 30 years or older were 9.6 times more likely to experience adverse outcomes than those born to younger mothers. (OR 9.6, p=0.03, 95% CI: 1.21-76.15), with statistically significant associations. Newborns given to Saudi women had 0.95 times the likelihood of having adverse outcomes (OR: 0.95, p=0.90, 95% CI: 0.10-7.35). Urban women were 0.65 times more likely than rural women to have babies with adverse outcomes. (OR 0.65, p=0.53, 95% CI: 0.16-2.54). Women with a family history of DM were 6.4 times more likely to have the disease than those without a family history of DM (OR 6.4, p=0.014, 95% CI: 1.45-28.58) and reported a significant statistical association. Women with four or more previous pregnancies had a 10.9-fold increased risk of adverse outcomes (OR 10.9, p=0.001, 95% CI: 2.84-42.7); this association was statistically significant. The odds ratio (OR) for adverse outcomes among infants born by cesarean section was 3.2 (p=0.26, 95% CI: 0.40-25.84).

**Table 4:** Logistic regression analysis for predictors and potential associations between GDM and newborn adverse neonatal outcomes (n=175). GDM: gestational diabetes mellitus

Variables	Odds ratio	p-Value	95% Confidence interval
Age (≥30 versus <30 years)	9.6	0.03	1.21-76.15
Nationality (Saudi versus non-Saudi)	0.95	0.90	0.10-7.35
Residence (urban versus rural)	0.65	0.53	0.16-2.54
Family history of DM (yes versus no)	6.4	0.014	1.45-28.58
Number of previous pregnancies (≥4 versus <4)	10.9	0.001	2.81-42.7
Mod of delivery (cesarean section versus vaginal)	3.2	0.26	0.40-25.84

## Discussion

This study addressed the health outcomes that influence predicting poor neonatal outcomes in newborns can be accomplished in several ways, all of which can be done through regular visits with a healthcare provider. This study found that the most common problems among the 12 babies born to mothers diagnosed with GDM who had bad outcomes were RDS, low birth weight, and macrosomia (affecting 25% of the babies each), followed by hypoglycemia (18%), and congenital heart abnormalities (7%), which is consistent with the reports of other studies [22-24]. Newborns with a low birth weight (less than 2.5 kg) are at a higher risk of developing health complications, such as respiratory distress syndrome and hypoglycemia. At the same time, the exact reasons for low-birth-weight infants are not fully understood and require further investigation.

This study’s findings are similar to previous research conducted in Saudi Arabia and elsewhere, which found that older mothers have a 9.6-fold higher risk of adverse infant outcomes than younger mothers. This proved that increased mother age plays a role in developing GDM problems [11,25-27]. According to the literature, this study discovered that the risk of adverse consequences is four times higher for women with a history of birth than for those with low parity. Higher blood sugar levels during pregnancy are linked to problems like premature delivery, macrosomia, and greater mortality, which may explain why numerous births are riskier [28,29]. Similar to the result by Esakoff et al., there was no significant association between GDM and poor infant outcomes in terms of characteristics such as Saudi nationality or residing in an urban region [30].

The current study revealed that mothers with GDM over 30 years had a 7.17-fold higher chance of having their newborns brought to the NICU, which agrees with the results of several other studies [6,31]. A recent study found that the rate of neonatal NICU admission and bad neonatal outcomes were higher in grand multiparity GDM cases. This is consistent with the findings of the present study, which indicate that women with GDM who have had more than four births have a 10.9-fold increased risk of NICU admission and poor neonatal outcomes compared to women with low parity [32].

This study confirms earlier findings that a family history of diabetes is a significant predictor of gestational diabetes mellitus (GDM) and the need for neonates to be admitted to the NICU. This can be explained by the high genetic susceptibility to diabetes mellitus in the Saudi population [33]. According to previous research, mothers with GDM are more likely to undergo cesarean sections, meaning their infants will spend more time in the NICU. This agrees with the current study’s results, which show a 3.38-fold increase in the chance of NICU admission for infants who were not born vaginally [34,35].

Identifying predictors of NICU admission and adverse outcomes related to gestational diabetes is important for improving outcomes for mothers and babies. Healthcare providers should work closely with pregnant women with gestational diabetes to ensure optimal glycemic control and monitor for other risk factors that may increase the likelihood of NICU admission or adverse outcomes.

It is important to find out what causes NICU admission and bad outcomes related to gestational diabetes so that the right steps can be taken to make things better. Although that is true, other confounding factors should be considered.

This study has multiple implications, including how the early identification of at-risk women can assist healthcare providers in implementing appropriate interventions and management strategies to prevent negative outcomes. Enhanced prenatal care can also reduce the likelihood of NICU admission and negative outcomes. In addition to raising awareness, this can enhance patient education and encourage women to seek medical care promptly. In addition to having cost implications, this can reduce healthcare expenditures. Future research may investigate additional predictors and interventions that may enhance outcomes for gestational diabetes-affected mothers and infants.

Limitations

The study was conducted in a hospital setting, which limits its generalizability to a wider population. Therefore, future studies with larger sample sizes should be conducted to improve the external validity of the findings. Moreover, future research should also examine other variables that may impact the incidence of GDM. It is important to note that the study is subject to limitations, such as incomplete medical records and inadequate record-keeping.

## Conclusions

Maternal factors significantly impact adverse infant outcomes and NICU admissions and thus need to be detected earlier and eliminated through regular visits to the health care provider. This highlights the importance of creating and applying an efficient and multidisciplinary strategy for managing GDM. This strategy dive by a team including PHC physicians, obstetricians, dietitians, health educators, social workers, nurses, and neonatologists. By working together as a team, these healthcare professionals can provide comprehensive care for women with GDM, helping to ensure adequate prenatal care for the health of both the mother and the newborn and reducing the risk of poor neonatal outcomes.
